# The Correlation between EGFR Mutation Status and DNA Content of Lung Adenocarcinoma Cells in Pleural Effusion

**DOI:** 10.7150/jca.38615

**Published:** 2020-02-06

**Authors:** Yun Du, Xiao Guo, Rui Wang, Yang Ma, Yan Zhang, Ying Liu, Lvli Dong, Juan Wu, Xiaokun Ji, Heng Wang

**Affiliations:** Department of Cytology, The Fourth Hospital of Hebei Medical University, Shijiazhuang, China

**Keywords:** Lung adenocarcinoma, EGFR mutation, DNA quantitative analysis

## Abstract

**Objectives**: Lung adenocarcinomas with or without epidermal growth factor receptor (EGFR) mutations have shown different drug effects against EGFR inhibitors. But it is not very clear if EGFR mutation status affects the biological behavior of lung adenocarcinoma, because tumor gene regulation is very complicated and can be affected by many factors. We aimed to explore if EGFR mutation status is related with tumor malignant degree by investigating the relevance of EGFR mutation status with DNA content and aneuploid peaks of lung adenocarcinoma cells in pleural fluids without using EGFR-TKIs.

**Materials and Methods**: 591 cases of lung adenocarcinoma patients in Hebei Tumor Hospital who had undergone EGFR gene detection and DNA quantitative analysis were collected from January 2012 to August 2018.They were divided into two groups: EGFR mutant group and non-mutant group. EGFR mutations were detected by Amplification Refractory Mutation System (ARMS) and ABI 7500 Fluorescence quantitative PCR with pleural effusions. DNA content and aneuploid peaks were detected by LD DNA image cytometry (DNA-ICM). Rank-sum test of SPSS 16 was used for statistical analysis.

**Results**: The maximum DI, the mean DI of the first 20 cells greater than 5C, the percentage of cells greater than 5C and the number of cells greater than 9C of the first 20 cells in the mutant group were all higher than those in the non-mutant group, having statistical significance (p<0.001); the peaks of aneuploid cells in the mutant group occurred more often than those in the non-mutant group, having statistical significance (p<0.001).

**Conclusions**: Our study has shown that advanced lung adenocarcinomas with EGFR-mutations had higher DI values, more aneuploid cells and more frequent aneuploid peaks compared with those without EGFR-mutations, suggesting that advanced lung adenocarcinomas with EGFR mutations are more aggressive than those without EGFR mutations.

## 1. Introduction

EGFR is a receptor tyrosine kinase that plays fundamental roles not only in physiology but also in cancer, its overexpression and/or mutations have been found in many cancers which may affect the development and progression of cancer 1-4. In recent years, EGFR targeted drugs have been widely used and achieved significant efficacy in lung adenocarcinoma, and have shown different effects in different EGFR mutation status 5-8. But the correlation and mechanism of EGFR mutation status with the biological behavior and prognosis of lung adenocarcinoma remain unclear and controversial, and can be interfered by tumor stage, EGFR-TKI medication and other factors.

DNA aneuploidy is an objective, molecular-based biomarker that reflects cancer genetic instability. Many studies have shown that the aneuploid cells in tumor tissues were associated with tumor malignancy and prognosis 9-14. But few researches evaluated the biological behavior of lung adenocarcinoma with or without EGFR mutations using DNA content detected by automatic DNA image cytometry. The aim of this study is to explore if and how EGFR mutation status affects the biological behavior of lung adenocarcinoma by detecting the DNA content and aneuploid peaks of advanced lung adenocarcinoma cells with or without EGFR mutations in the absence of EGFR-TKIs.

## 2. Materials and Methods

### 2.1. Materials

591 cases of advanced lung adenocarcinoma patients with pleural effusion in Hebei Tumor Hospital who had undergone EGFR gene detection and DNA quantitative analysis were collected from January 2012 to August 2018. All the patients met our strict inclusion criteria : (1) They were diagnosed with lung adenocarcinoma by two skillful cytologists; (2) They were never received any treatment prior to admission; (3) All of them were in stage IV according to the UICC/AJCC TNM staging system; (4) They and their families agreed to participate in the study and signed the informed consent form; (5) They received EGFR gene mutations test and DNA quantitative analysis; (6) They did not combine other tumors; (7) This study was approved by the Hospital Ethics Committee. They were divided into two groups: EGFR mutant group and non-mutant group. EGFR mutations were detected by Amplification Refractory Mutation System (ARMS) and ABI 7500 Fluorescence quantitative PCR with pleural effusions. DNA content and aneuploid peaks were detected by LD DNA image cytometry (LD DNA-ICM, Landing Med Tech, Wuhan). Clinical and pathological data of all the patients were obtained from the medical records and telephone follow-ups from patients or their families. Rank-sum test of SPSS 16 was used for statistical analysis.

### 2.2. Sampling Method

**Figure 1:**
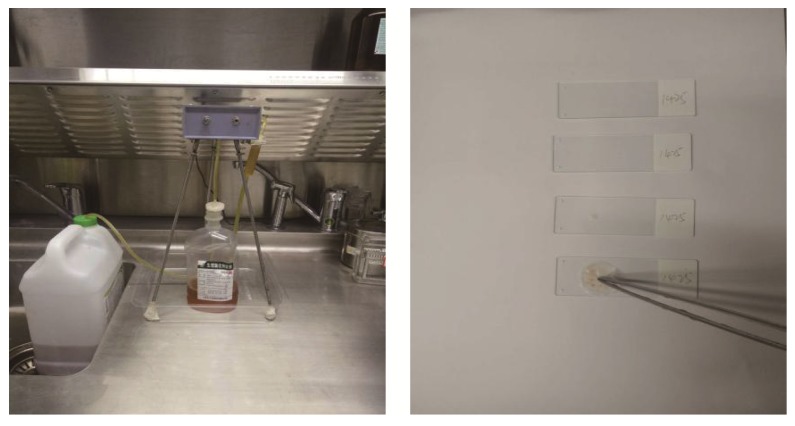
Sampling method

2.2.1. As shown in Figure 1, first we used a disposable cell collector to extract and filter part of the pleural effusion(100~300ml), which mechanism was to use a filter membrane with different-size micropores to trap and enrich tumour cells, then the enriched cells were uniformly smeared on 4 ordinary slides and 8 antistripping slides: two of the four ordinary slides were fixed in 95% alcohol for routine diagnosis; two of the four ordinary slides were fixed in anhydrous ethanol for DNA quantitative analysis; eight anti-stripping slides were fixed in 95% ethanol for immunocytochemical staining.

2.2.2. The remaining pleural effusion was centrifuged for 2 minutes at 1800 r/min using a 15ml tube several times until it was used up, every time the supernatant was discarded and the bottom sediment was retained, finally the total sediment was stored in - 20 ℃ refrigerator for EGFR gene detection.

### 2.3. EGFR mutation detection

2.3.1. We used AmoyDx DNA extraction kit to extract DNA and used Denovix DS-11 ultraviolet spectrophotometer to detect DNA concentration and purity. OD260/OD280 was within 1.8 ~ 2.0, the recommended concentration of the DNA from fresh specimens was 0.4~1ng/μl.

2.3.2. We used ARMS and ABI 7500 Fluorescence quantitative PCR to detect EGFR gene mutation. The procedures were carried out according to AmoyDx EGFR gene mutation detection kit.

### 2.4. DNA quantitative analysis

We used LD DNA-ICM to detect DNA content, aneuploid cells and aneuploid peaks, which included an Olympus BX41 microscope, a Wave NP370D2 server, a ueyeM2240 camera, an automatic control platform, and a cell image analysis software. Its mechanism was to determine the nucleus DNA content by measuring the integrated optical density (IOD) of the stained nucleus DNA, using normal epithelial cells and lymphocytes on the same slide as control. The reference cell coefficient of variation (CV, CV= standard deviation / mean value × 100%) was less than 5%.

DNA Index (DI) was used to represent DNA content: DI= DNA IOD of the tested cell /DNA IOD average of normal cell 15. If the measured cell was in the G0/G1 phase, its IOD was very close to the average IOD of normal cells, so the DI was 1, that was 2C (1C was half of the DNA content of normal G0/G1 cells, so G0/G1 cells were 2C cells, namely diploid cells); When the cell was in G2/M phase, its IOD was about twice the IOD average of normal cells, so DI was 2, that was 4C, and so on. Aneuploid cell peaks included: ①single peak, which could appear in different positions, mostly between 2C ~ 4C; ② double peaks, which could appear in a variety of tumors, DI of the second peak was often twice that of the first peak; ③multi-peaks, the histogram showed different heights like Manhattan houses, and the appearance of multiple peaks indicated that the chromosome structure of the tumor was very unstable 15.

**Figure 2:**
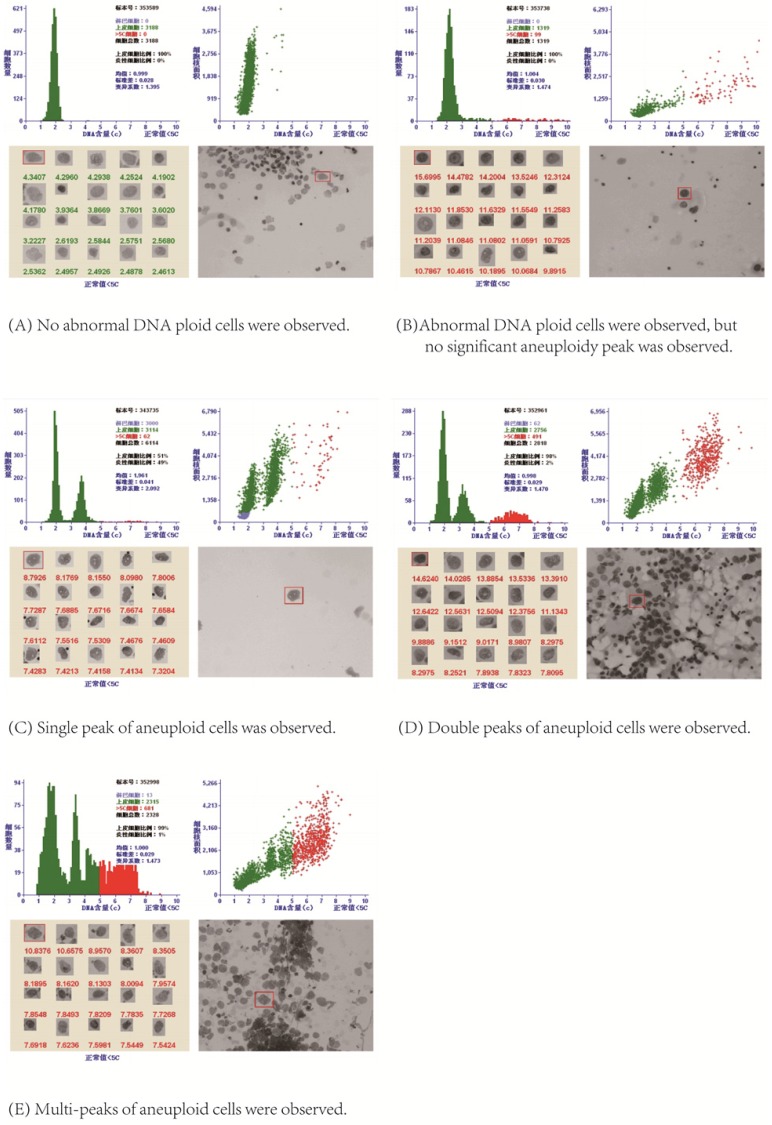
DNA quantitative analysis.

After stained by Feulgen staining, all the nuclei on the slide were scanned on the automatic LD DNA-ICM. When the DI values of aneuploid cells formed a peak between 1.1~1.9; or there were more than 3 cells with DI larger than 2.5, namely larger than 5C; or the number of cells with DI=2 accounted for more than 10% of the total tested cells, these were used as positive indicators for DNA quantitative diagnosis of cancerous pleural /peritoneal effusion 15. As shown in Figure 2, LD DNA-ICM could show the total number of epithelial cells, the number of cells greater than 5C, the C values of top 20 cells with the highest DNA content and aneuploid peaks in the report.

## 3. Results

3.1 In 591 patients analyzed, the median age was 66 years old, ranging from 28 to 93 years. 226 patients were older than 66 years old (38.2%), and 365 patients were less than or equal to 66 years old (61.8%). As for gender, 257 (43.5%) were men and 334 (56.5%) were women. 213 of the 591 patients (36.0%) had smoking history and 378 (64.0%) had not smoking history. All the patients were in advanced stage with pleural effusion and inoperable, so the histological specimens could not be obtained. Therefore, we could only diagnose lung adenocarcinoma by HE-staining cytological smear and immunocytochemistry, unable to further classify histopathological subtypes. All of them were stage IV according to the UICC/AJCC TNM staging system. We analyzed the relationship between EGFR mutations and clinicopathologic features, and found EGFR mutation status was correlated with gender (p=0.034) and smoking habits (p=0.007), but not correlated with age (p=0.483). The clinicopathological characteristics of those patients were summarized in Table 1.

**Figure 3:**
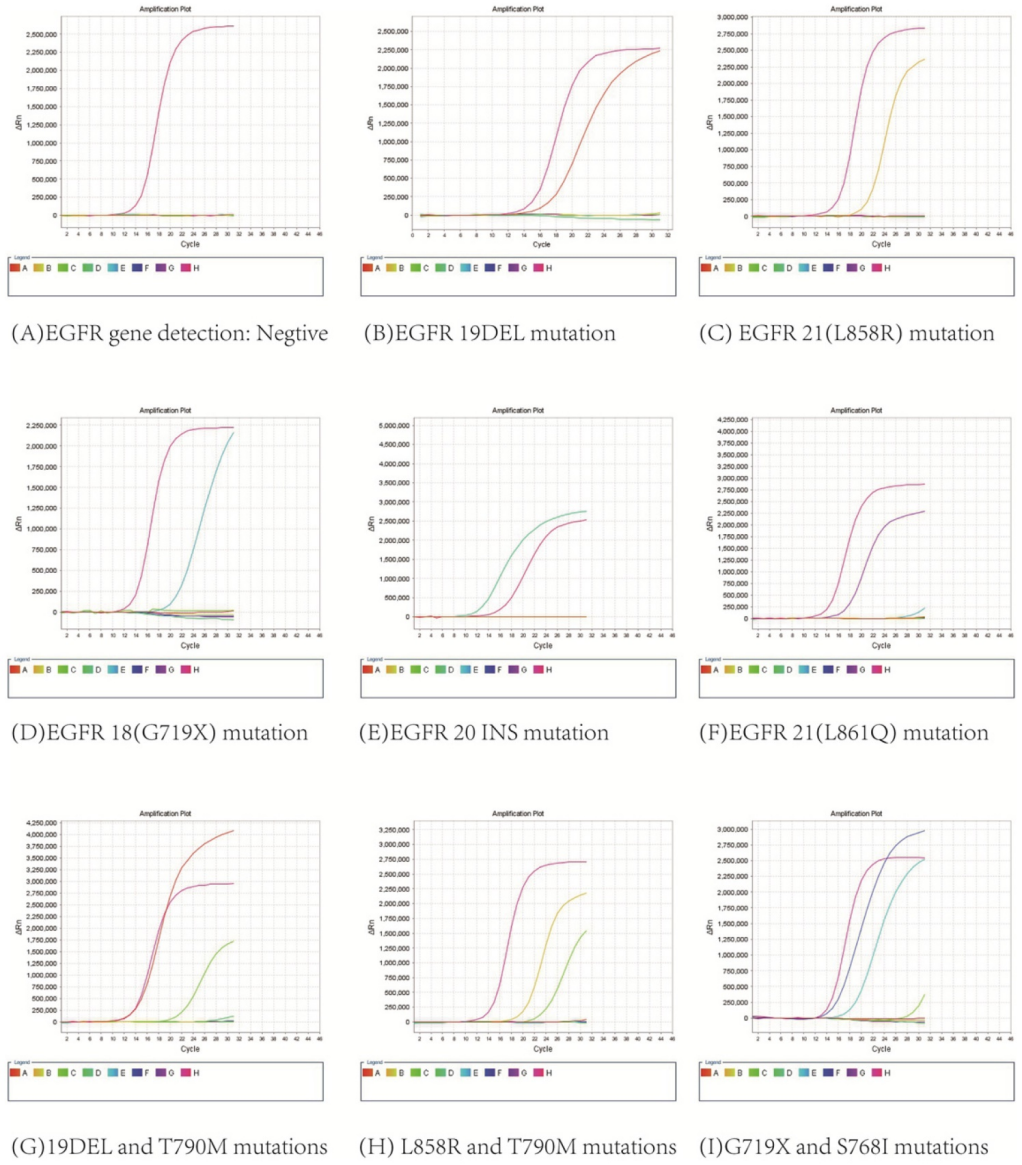
EGFR gene mutation results.

3.2. All 591 patients were diagnosed with lung adenocarcinoma by HE staining and immunocytochemical staining, and EGFR gene detection and DNA quantitative analysis were successfully performed with their pleural effusions. As shown in Figure 3, EGFR mutations were found in 335 patients, including 9 with G719X mutation, 130 with 19DEL mutation, 4 with 20INS mutation, 155 with L858R mutation, 1 with L861Q mutation, 18 with combined mutations of 19DEL and T790M, 14 with combined mutations of L858R and T790M, 2 with combined mutations of 19DEL and L858R, 2 with combined mutations of G719X and S768I, and there were 256 patients without mutations.

3.3. Table 2 shows the comparison of DNA quantitative analysis between mutant group and non-mutant group. The maximum DI, the mean DI of the first 20 cells greater than 5C, the percentage of cells greater than 5C, and the number of cells greater than 9C of the first 20 cells in the mutant group were all higher than those in the non-mutant group, having statistical significance (p<0.001).

Table 3 shows the comparison of DNA aneuploid cell peaks between the mutant group and the non-mutant group. The peaks of aneuploid cells in the mutant group occurred more often than those in the non-mutant group, having statistical significance (p<0.001).

3.4. Table 4 shows the comparison of DNA quantitative analysis between 19DEL, L858R and non-mutant group. There were 150 cases in the 19DEL group (including 19DEL, 19DEL and T790M, 19DEL and L858R), 171 cases in the L858R group (including L858R, L858R and T790M, 19DEL and L858R), and 256 cases in non-mutant group. The maximum DI, the mean DI of the first 20 cells greater than 5C , the percentage of cells greater than 5C and the number of cells greater than 9C of the first 20 cells were compared in three groups, showing statistical significance: L858R > 19DEL > non-mutant group (p<0.05); Table 5 shows the comparison of DNA aneuploid cell peaks among three groups: the aneuploid peaks showed a statistically significant difference in three groups(p < 0.001), but there was no statistical difference between L858R group and 19DEL group when compared between two groups respectively.

## 4. Discussion

It is well known that lung adenocarcinomas with or without EGFR mutations have shown different drug effects against EGFR inhibitors. But EGFR is an oncogene; the abnormality of its related pathways is closely associated with tumor development, invasion, metastasis and drug resistance 1-4, 16-19. Up till now it remains unclear and controversial whether there is a difference in malignant degree and prognosis between patients with EGFR mutations and those without mutations. Some studies have reported that patients with EGFR mutations were associated with a better prognosis compared with those without EGFR mutations, but most of them were based on the use of EGFR-TKI 20-23. Two studies from Tetsuya Isaka et al 24 and Masaya Yotsukura et al 25 have reported patients with EGFR mutations in operable early-stage lung adenocarcinoma had a better RFS because of its higher incidence of lepidic growth pattern, adenocarcinoma in situ and minimally invasive adenocarcinoma*,* which rarely recur after resection of the lung when compared with those without EGFR mutations, but there was no significant difference in OS. However whether the histological subtype of lung adenocarcinoma is a prognostic factor remains controversial 26-28. In contrast, some researchers 29, 30 have found that lung adenocarcinomas harboring some EGFR mutations exhibited increased cancer cell invasive ability and promoted malignant pleural effusion formation, and Zhu et al 31 have reported that Mt has a higher TP53 mutation rate and a higher Ki67 expression compared with Wt, suggesting that Mt is more aggressive. So it is controversial and unclear if and how different EGFR mutation status affects the biological behavior of lung adenocarcinoma without using EGFR-TKI. We want to find an objective and molecular-based indicator to examine the effect of EGFR mutation status on the biological behavior of lung adenocarcinoma. Many studies 32-37 have demonstrated that DNA aneuploidy represents chromosomal instability and is the most common genetic abnormality in malignant cells related to the development of tumor. Njølstad et al 38 investigated DNA ploidy in curettage specimens of 785 endometrial carcinomas, and found non-diploid curettage is significantly associated with aggressive clinicopathological phenotype, lymph node metastasis, and poor survival in endometrial cancer. Maounis et al 39 reported that DNA ploidy, as determined by image analysis, provided an independent prognostic parameter for patients with NSCLC and thus, could be used to identify a subset of patients with more aggressive tumors. Other researches 40, 41 have demonstrated high aneuploid DNA content was related to higher incidence of vessel invasion and lymph node metastasis. It has been identified by many studies that DNA content or DNA aneuploidy is an independent prognostic factor in many cancers 9-14, 42-49. So we chose DNA as an objective genetic indicator to evaluate the biological behavior of lung adenocarcinoma in different EGFR mutation status.

The results of this study have shown that the mutant group had higher DI values, more aneuploid cells and more frequent aneuploid peaks compared with the non-mutant group; L858R group had higher DNA content, more aneuploid cells and peaks, suggesting more genetic instability and malignancy when compared with 19Del group, consistent with previous studies 29, 50.

Based on the previous researches above mentioned, our study suggests that advanced lung adenocarcinomas with EGFR mutations are more aggressive than those without EGFR mutations.

Our study had several limitations. First, follow-up and survival analysis were not performed, so we are not sure if EGFR mutation status in advanced lung adenocarcinoma is an independent factor affecting prognosis. Second, It is not rigorous enough to evaluate the malignancy of tumor only by DNA content, although most reports have demonstrated that high DNA content, more aneuploid cells are closely related to tumor malignancy and poor prognosis, but a few studies have shown that the level of DNA content and the number of aneuploid cells are not independent factors affecting prognosis 51.

The effect of EGFR mutation status on tumor malignancy and prognosis is interfered by many factors, such as tumor stage and treatment method, etc. Our next study is to further investigate if EGFR mutation status and DNA aneuploidy are independent factors affecting the prognosis and survival of patients with lung adenocarcinomas by supplementing and refining follow-up data, and to design basic experiments to test whether different levels of DNA aneuploidy can change the proliferation, invasion and metastasis ability of lung adenocarcinoma cells.

## Conclusion

This study has shown that there were significant differences in the value of DNA content, the number of aneuploid cells and the frequency of aneuploid peaks between EGFR mutant group and non-mutant group, the mutant group had higher DI values, more aneuploid cells and more frequent aneuploid peaks compared with the non-mutant group, suggesting that advanced lung adenocarcinomas with EGFR mutations were more aggressive than those without EGFR mutations.

## Figures and Tables

**Table 1:** Clinicopathological features of patients with lung adenocarcinoma.

Group (EGFR)	WT number	MT number	Positive rate (%)	χ^2^	P value
Age (yr)				0.492	0.483
≤66	154	211	57.8%		
>66	102	124	54.8%		
Gender				4.506	0.034
Male	124	133	51.7%		
Female	132	202	60.4%		
Smoking				7.403	0.007
Smoker	108	105	49.3%		
Non-smoker	148	230	60.8%		
TNM Stage					
IV	256	335	56.7%		
Cytopathology					
adenocarcinoma	256	335	56.7%		

**Table 2:** The comparison of DNA quantitative analysis between the mutant group and the non-mutant group

**EGFR result**	**The maximum DI**				**The mean DI of the first 20 cells greater than 5C**			
	**Median**	**Mean rank**	**T**	**p**	**Median**	**Mean rank**	**T**	**p**
Mutant group	6.1224	317.37	35719.50	<0.001	4.3987	323.35	33719.00	<0.001
Non-mutant group	5.5992	268.03			3.9988	260.21		
		
**EGFR result**	**The percentage of cells greater than 5C**				**The number of cells greater than 9C**			
	**Median**	**Mean rank**	**T**	**p**	**Median**	**Mean rank**	**T**	**p**
Mutant group	3.7521	330.82	31214.50	<0.001	7	320.83	34561.50	<0.001
Non-mutant group	1.7445	250.43			3	263.51		

**Table 3:** The comparison of DNA aneuploid cell peaks between the mutant group and the non-mutant group

	Single peak	Double peak	Multi-peak	None peak	Mean rank	T	p
Mutant group	124	38	47	126	320.14	34793.50	<0.001
Non-mutant group	59	15	33	149	264.41		

**Table 4:** The comparison of DNA quantitative analysis among the 19DEL group, L858R group and the non-mutant group

**EGFR result**	**The percentage of cells greater than 5C**				**The number of cells greater than 9C**			
	**Median**	**Mean rank**	**H**	**p**	**Median**	**Mean rank**	**H**	**p**
19DEL	2.9510	296.1	42.00	<0.001	5	293.51	23.48	<0.001
L858R	5.9214	350.17			9	334.70		
Non-mutant group	1.7445	243.94			3	255.83		
		
**EGFR result**	**The maximum DI**				**The mean DI of the first 20 cells greater than 5C**			
	**Median**	**Mean rank**	**H**	**p**	**Median**	**Mean rank**	**H**	**p**
19DEL	6.1449	304.70	12.82	0.002	4.2845	291.46	27.86	<0.001
L858R	6.0887	316.26			4.5405	340.2		
Non-mutant group	5.5992	261.59			3.9988	253.36		

**Table 5:** The comparison of DNA aneuploid cell peaks among the 19DEL group, L858R group and the non-mutant group

	Single peak	Double peak	Multi-peak	None peak	Mean rank	H	p
19DEL	61	15	15	59	300.84	18.93	<0.001
L858R	58	22	29	62	323.52		
Non-mutant group	59	15	33	149	259.00		
